# Interventions to support fellowship application success among predoctoral physician-scientists

**DOI:** 10.1172/jci.insight.175857

**Published:** 2024-03-08

**Authors:** Reiko Maki Fitzsimonds, Fred S. Gorelick, Barbara I. Kazmierczak

**Affiliations:** 1 MD-PhD Program,; 2 Department of Medicine (Digestive Diseases) and Cell Biology, and; 3 Department of Medicine (Infectious Diseases) and Microbial Pathogenesis, Yale School of Medicine, New Haven, Connecticut, USA.

## Abstract

A critical element of physician-scientist training is the development and practice of core competencies that promote success in research careers. The ability to develop compelling training and research proposals is one such foundational skill. The NIH Ruth L. Kirschstein National Research Service Award (NRSA) individual fellowship for dual-degree students (F30, F31, or F31-Diversity) creates an ideal opportunity to provide formal instruction in grant-writing skills to physician-scientists early in training. In the guided process of preparing a predoctoral fellowship application, students learn to formulate clear short- and long-term research and training goals; construct a comprehensive, well-reasoned, and rigorous proposal; become familiar with funding agency priorities; and gain strategic insights into the peer review system. Beyond building scientific writing skills, the application process for an NRSA F30 or F31 is an opportunity for trainees to strengthen mentor-mentee relationships, identify learning opportunities key to their scientific development, and build effective research and mentoring teams. These skills also apply to developing future postdoctoral mentored K applications or faculty research program grants. Here, we outline key features of the structured proposal development training developed for students in the Yale MD-PhD Program and review outcomes associated with its implementation.

## Introduction

Physician-scientists are a critical part of the biomedical workforce, uniquely positioned to recognize and address unmet needs in the diagnosis and care of patients. Much effort has been devoted into developing robust dual-degree training programs that prepare trainees to practice medicine and lead rigorous scientific research (1, 2). Whether such a career is realized in an academic medical center, a biotech or pharmaceutical company, or at the NIH, the ability to identify a significant knowledge gap and develop a research plan that addresses that gap is fundamental in order for physician-scientists to obtain the necessary resources and support for their work. Grant writing skills, therefore, represent an essential competency for any trainee who aspires to pursue a career as a physician-scientist. Thus, we conclude that MD-PhD training programs should ensure that students have adequate training and opportunities to develop such skills as part of their core curricula.

Although many predoctoral programs teach scientific writing skills through mechanisms such as qualifying examination papers that follow the structure of a research proposal, there remains a need for training that systematically addresses specific aspects of developing a successful grant proposal. The emphasis of individual fellowship applications on training rather than research, per se, encourages applicants to define the learning activities, experiences, and opportunities most aligned with their long-term goals as nascent physician-scientists. The need to support their proposed research with information about resources, mentors, collaborators, and infrastructure gives applicants insight into the thorough preparation needed to become an independent investigator. Likewise, required elements outlining rigor and reproducibility compel trainees both to refine their experimental and analytic approaches and to identify the need for additional training in these areas early in their careers. A curriculum that provides detailed explanations and resources for all the steps in proposal preparation, submission, review, and resubmission at an early stage of training is well suited for developing the skills, insights, and confidence needed for success with subsequent career development (K award) and research program grant (RPG) proposals (1–3). This experience should also provide a unique foundation for trainees to serve as mentors to their own future trainees.

As a platform for developing these skills, over the past decade, the MD-PhD Program at Yale has incorporated the preparation (and submission) of Ruth L. Kirschstein National Research Service Award (NRSA) individual predoctoral fellowship applications (F30, F31, or F31-Diversity) into our overall training curriculum. This effort has significantly increased grant proposal submissions. There are many potential barriers to developing a successful predoctoral fellowship application, ranging from inadequate mentorship, lack of administrative and institutional support, ambiguities about the application and review processes, and personal motivations. We have developed effective interventions that address these activation barriers, and it is now an expectation of the Yale MD-PhD Program that all eligible trainees submit an F30, F31, or F31-Diversity application during training. Anecdotally, students report experiencing less anxiety and more confidence and enthusiasm to develop NRSA applications as part of, or in conjunction with, preparing to qualify for PhD candidacy.

The Yale MD-PhD program currently organizes 2-hour workshops coinciding with the NIH F30 and F31 application cycles in February and October of each year and offers a four-session course on proposal development each June that is required for all students entering their fourth year in the program. Although other resources that support the preparation of predoctoral fellowship applications are available at Yale, the MD-PhD Program’s workshops and a course emphasize those aspects of research, training, and professional development unique to NRSA applications submitted by physician-scientist trainees.

Beyond requiring participation in workshops that provide an overview of the entire proposal-development process, two critical interventions have significantly lowered activation barriers and encouraged students to confront the challenge of developing and submitting NRSA applications. First, students receive early one-on-one coaching and advice on crafting their research and training goals with program faculty who have first-hand experience with the NIH fellowship peer review process. Second, several administrative innovations have helped students and their mentors to anticipate and manage the workload and timeline of the NRSA preparation process which simply cannot be rushed. These innovations are as follows.

### An “intent to apply” survey.

Four months in advance of each NIH NRSA fellowship deadline, a survey (Supplemental Appendix 1; supplemental material available online with this article; https://doi.org/10.1172/jci.insight.175857DS1) is sent to all eligible third- and fourth-year MD-PhD students. Completion of the survey automatically notifies the student’s mentor of the mechanism (F30, F31, or F31-Diversity) and NIH cycle (April, August, or December), details their responsibilities as sponsors, and requests affirmation of their commitment to actively engage in the proposal writing process with the student. Faculty have been very responsive to this notification. Junior faculty or those who have not previously had a student submit an F application use this opportunity to request detailed guidance on expectations and responsibilities of an F application sponsor.

### An extensive online presubmission survey.

Students are given access to an online presubmission survey (Supplemental Appendix 2) once they indicate their “intent to apply.” The survey ensures that all the information necessary to complete the SF424 (R&R) Form is gathered in a timely fashion by the student and that the final assembly of the application itself by the program’s grants administrator is accurate and efficient. Confirmation that the regulatory requirements of Yale University (i.e., Conflict of Interest Disclosure form, Sponsored Projects Administration Training, Yale NRSA Assurance of Compliance form) are completed in advance of the submission date is also obtained. Questions about human and animal subjects include links to important NIH information, ensuring that students fully understand and comply with the guidelines for the responsible conduct of research. Detailed instructions for students to request correct and timely submission of letters of reference through eRA Commons are included in the survey. Finally, examples of information required for facilities and other resources, equipment, key biological and/or chemical resources, and other required attachments are available to all students include the following. (a) A detailed checklist for document preparation, file naming convention, and sharing with program administrator. The sheer number of required sections to complete the SF424 (R&R) Form is daunting, particularly for a student who has never prepared a complex application. Students are often surprised to realize how much time and effort are required beyond writing the research and training sections. By providing examples of each required section and allowing students to upload finished documents (in Word and PDF formats) over an extended period of time in advance of the final assembly appointment with the program’s grants administrator, we have significantly lowered the activation energy for students to tackle the extensive checklist. (b) A centralized resource hub. Resources and guides for preparing an NRSA fellowship application are centrally located in a student folder on a secure cloud server, as well as online on our program intranet website (available only to current students). The most accessed resource is the collection of successful applications (provided with permission of the applicant) where students can review examples of well-written proposals and timelines for their implementation. Also included in the hub are the current program announcements with critical sections highlighted, NIH guidelines for reviewers, PowerPoint decks of grant-writing workshops, tips for contacting the program officer, guidelines for mentors writing sponsor statements, responsible conduct of research training syllabi, articles on rigor and reproducibility, and more.

We have examined the outcomes associated with our program’s proposal development training, with the goal of understanding which innovations and improvements have been most effective for trainees. Here we demonstrate significant increases in the overall number of applications submitted and funded over the past 20 years and discuss the key interventions that may have contributed to these positive outcomes.

## Results

### NIH investment in predoctoral physician-scientist training.

The NRSA F30 awards (current NIH-funding opportunity announcement PA-23-260; formerly PA-21-049) provide up to six years of support for students in dual-degree programs receiving institutional support from the NIH’s National Institute of General Medical Sciences (NIGMS) through the Medical Scientist Training Program (MSTP). A separate F30 funding opportunity announcement (PA-23-261) exists to fund dual-degree predoctoral trainees at institutions that do not have MSTP support.

**Figure 1:**
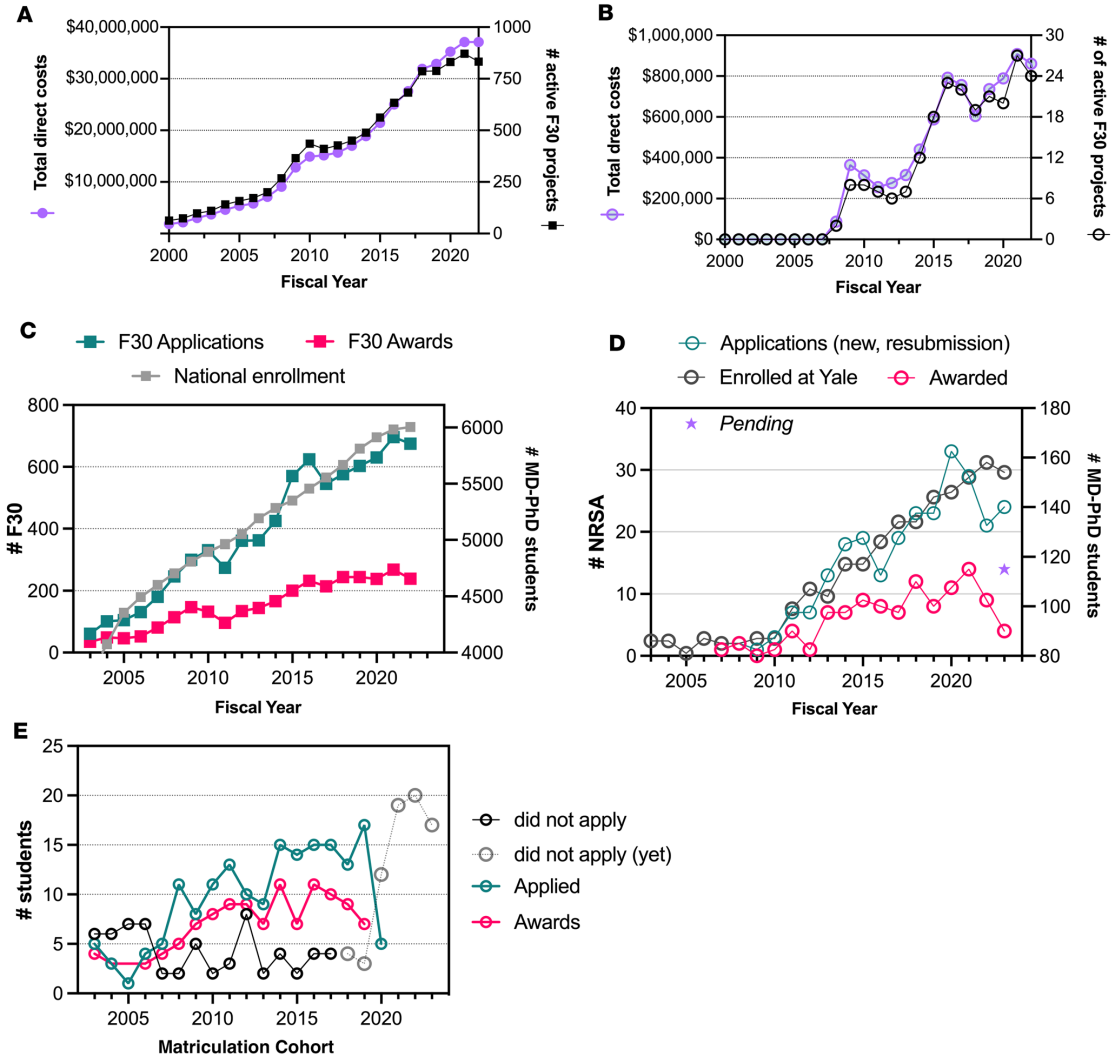
Comparing NIH F30 application and award numbers to Yale MD-PhD Program submission and award data. (**A** and **B**) Data from the NIH RePORTER using the advanced projects search “project details” category F30 for FY2000–FY2003 to plot (**A**) the number (black) and total annual expenditure (purple) for F30 projects supported across all participating NIH Institutes (**A**) and all F30 projects awarded to the “organization” Yale University (**B**). (**C**) Data from the NIH Data Book for the number of F30 applications received (green squares) and awards made (pink squares) across all participating NIH institutes; AAMC FACTS tables provided the Total MD-PhD Enrollment by U.S. Medical School. (**D**) The Yale University Research Enterprise Operations Sponsored Awards database data for all F30, F31-Diversity, and F31 (MD-PhD students only) applications submitted in FY2003–FY2023 follows a similar rate of growth as the increasing enrollment into the Yale MD-PhD Program. (**E**) The number of students in each matriculation cohort (MD-PhD students entering each year) who submitted (green circles) or were awarded (pink circles) an NRSA (F30 or F31). Since 2012, when the workshops were introduced, the goal has been to have all eligible students prepare and submit an NRSA fellowship application, minimizing the number who did not apply (black circles).

According to the NIH Research Portfolio Online Reporting Tools Expenditures and Results (RePORTER), the first predoctoral NRSA Individual Fellowships specifically for MD-PhD trainees (F30) were awarded in 1990 by the National Institutes of Alcohol Abuse & Alcoholism (NIAAA) and the National Institute of Mental Health (NIMH), with 7 fellowships totaling $157,091. Figure 1A describes data obtained from the NIH RePORTER Advanced Project Search (4) showing a steady increase in the number and funding of F30 projects between 2000 and 2022, culminating in 833 awards spanning 17 NIH Centers and Institutes, amounting to more than $37 million in total costs in fiscal year 2022 (FY2022). The corresponding number of F30 projects and total direct costs through the F30 mechanism awarded to Yale University in FY2000–FY2022 is shown in Figure 1B, with 24 active F30 projects totaling $860,791 in FY2022. Figure 1C shows that the steady increase in the number of F30 applications received by NIH tracks with the increase in national MD-PhD student enrollment (5–8), indicating that the proportion of students applying for these awards remains constant (between 10% and 12% of all enrolled students since 2015). The term “national enrollment” includes students in all years of an average 8-year MD-PhD training program, < 0.5% of whom are ineligible for federal funding mechanisms (9). In addition to the F30 award mechanism, eligible MD-PhD students may apply for F31 or F31-Diversity NRSA Predoctoral Individual Fellowships, which support PhD training. The F31-Diversity fellowship mechanism (PA 23-271) for MD-PhD students supports up to six years of both research and clinical training, whereas the F31 fellowship mechanism (PA 23-271) supports up to five years of mentored research training while conducting dissertation research. However, as the NIH RePORTER database does not specify which students supported by an F31 or F31-Diversity are dual-degree candidates; only F30 data are shown in Figure 1, A and C. For Figure 1, D and E, we combined the F30 and F31 (referred to as F30/F31) data to show that the steady increase in the number of NRSA applications follows the increase in total MD-PhD student enrollment at Yale, with a slightly higher proportion of students (14%) overall submitting applications compared with the national data in Figure 1C. Figure 1E presents the applications and awards data by matriculation cohort rather than by FY, showing the actual number of students who applied, who were funded, and who did not submit an NRSA application.

Figure 1D shows a steep increase in awards beginning in 2012 (2008 matriculation cohort in Figure 1E) that corresponds with the introduction of formalized grant-writing workshops for MD-PhD students. A drop in the number of submissions in FY2017 anecdotally corresponds to a one-year hiatus in workshop offerings, and a smaller decline in 2022 may reflect the lack of in-person workshops and coaching during the COVID-19 pandemic. These dips in submissions do not correspond to a drop in the number of eligible applicants, suggesting the possibility that the workshop offerings may drive or affect the number of student submissions. While the percentage of enrolled students applying for and being awarded F30 fellowships at Yale are slightly higher than national percentages, without a comprehensive survey of other MD-PhD programs to determine the availability of grant-writing workshops and extent of support, a causal relationship between fellowship success and grant-writing courses remains unclear. However, the adoption of a formalized curriculum to provide MD-PhD students with foundational grant-writing skills remains an essential component of our overall effort to provide rigorous training in leadership and research management for physician-scientists.

### Extramural funding outcomes of the Yale MD-PhD program.

**Figure 2:**
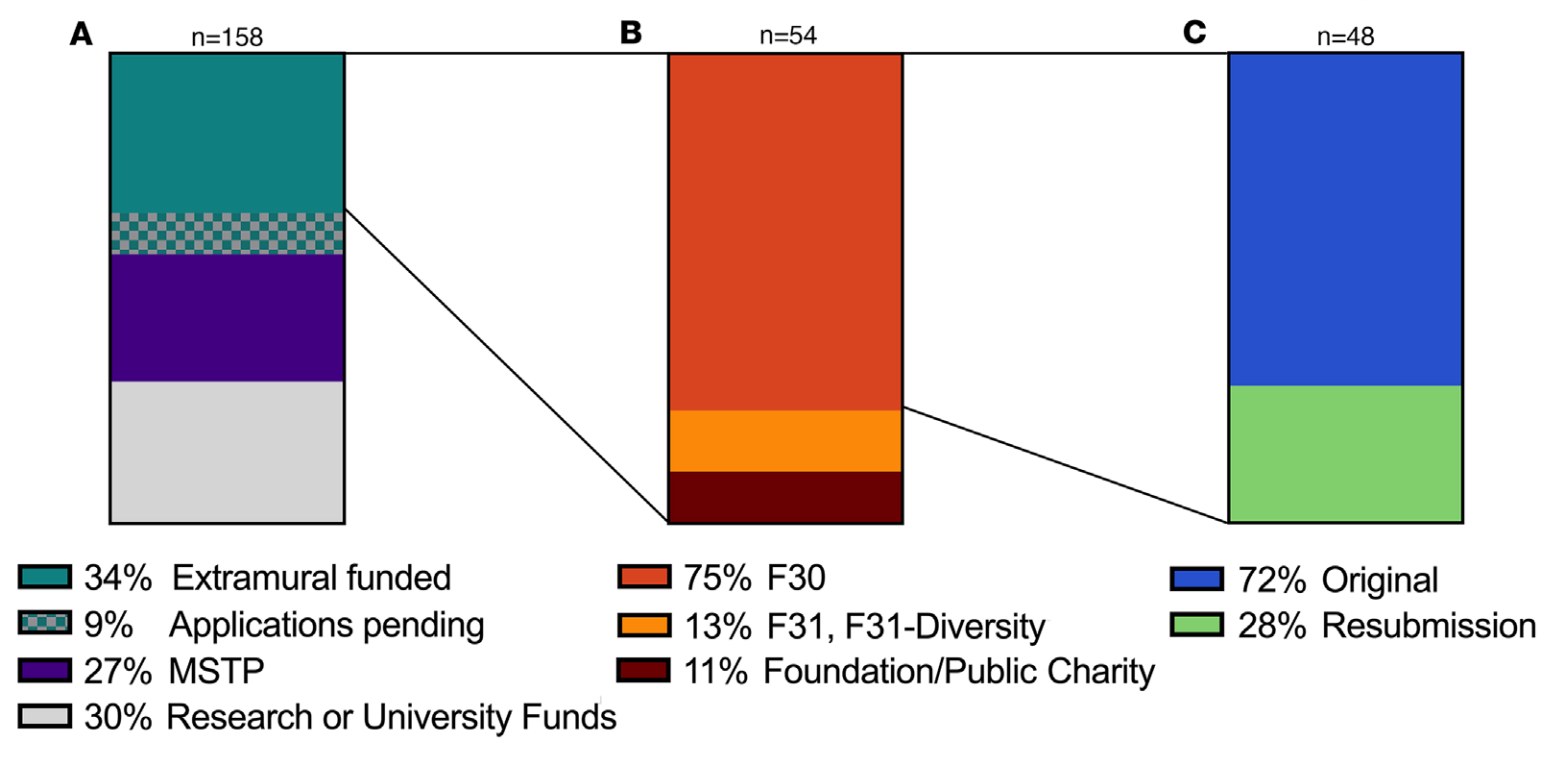
Sources of support for Yale MD-PhD students. (**A**) 158 current (academic year 2022–2023) students in the MD-PhD Program are supported by funds from investigator research grants and university funds (30% + 9% students with pending NIH fellowship applications), the NIGMS MSTP training grant (27%), and from extramural fellowships (34%). (**B**) Current students who are or have been supported by an NRSA F30 award (75%), an NRSA F31 award (13%), or private foundation (Robert Wood Johnson, The Wenner-Gren Foundation for Anthropological Research, Inc., Daisy and Paul Soros Foundation) or public charity (American Heart Association) (11%). (**C**) Of the NRSA F30 and F31 fellowships awarded, the majority (72%) is successful with the original application whereas 28% are awarded upon resubmission.

As of academic year 2022–2023, 158 currently enrolled students in the Yale MD-PhD Program are supported by several sources, as shown in Figure 2A. The MSTP training grant and its supplements are primary sources of support for 43 MD-PhD students (27%) who are mostly in the first two years of training. Once students affiliate with their doctoral research department and advisor, the stipend and tuition support source shifts primarily to the dissertation sponsor’s federal and nonfederal research funding and university funds. A positive outcome of 10 years of grant-writing training is that over one-third of eligible students currently enrolled (54 students; 34%) have or have had extramural fellowship funding, and an additional 14 (9%) have submitted extramural funding applications with award decisions currently pending. As shown in Figure 2B, the majority of currently enrolled students with extramural funding received F30 (41 students; 75%) compared with F31 and F31-Diversity (7 students; 13%) awards. The remaining students (6 students; 11%) are supported by fellowships from foundations and public charities, such as the Paul & Daisy Soros Foundation, the Robert Wood Johnson Foundation, and the American Heart Association. A second positive outcome of support and rigorous training is that 72% (34 of 48) of the current NRSA (F30, F31, F31-Diversity) fellowship applications were awarded on the first submission, with the remaining applications requiring resubmission (Figure 2C).

Seventeen of 20 total students in the 2019 matriculation cohort have prepared and submitted F30 or F31 applications; of these, seven have been awarded fellowships thus far. Typical reasons for not applying include ineligibility due to citizenship, a thesis project in the humanities that is not aligned with NIH funding priorities, or delays in starting dissertation research. Students who miss the eligibility window of 48 months after matriculation for F30 submission are highly encouraged to prepare F31 applications within their fifth year. Students in matriculation years 2020–2022 are currently preparing to apply or have not yet initiated the application process. The MD-PhD Program’s explicit goal is that all eligible students submit F30/F31 fellowship applications. This goal will be achieved through the regular grant-writing workshops coinciding with NIH submission deadlines that are open to all students, through active encouragement and regular reminders about deadlines and requirements, through one-on-one advising, as well as the introduction of a required 4-week Proposal Development bootcamp in the summer before the beginning of a student’s fourth year in the program.

### When to apply for NRSA fellowships.

**Figure 3:**
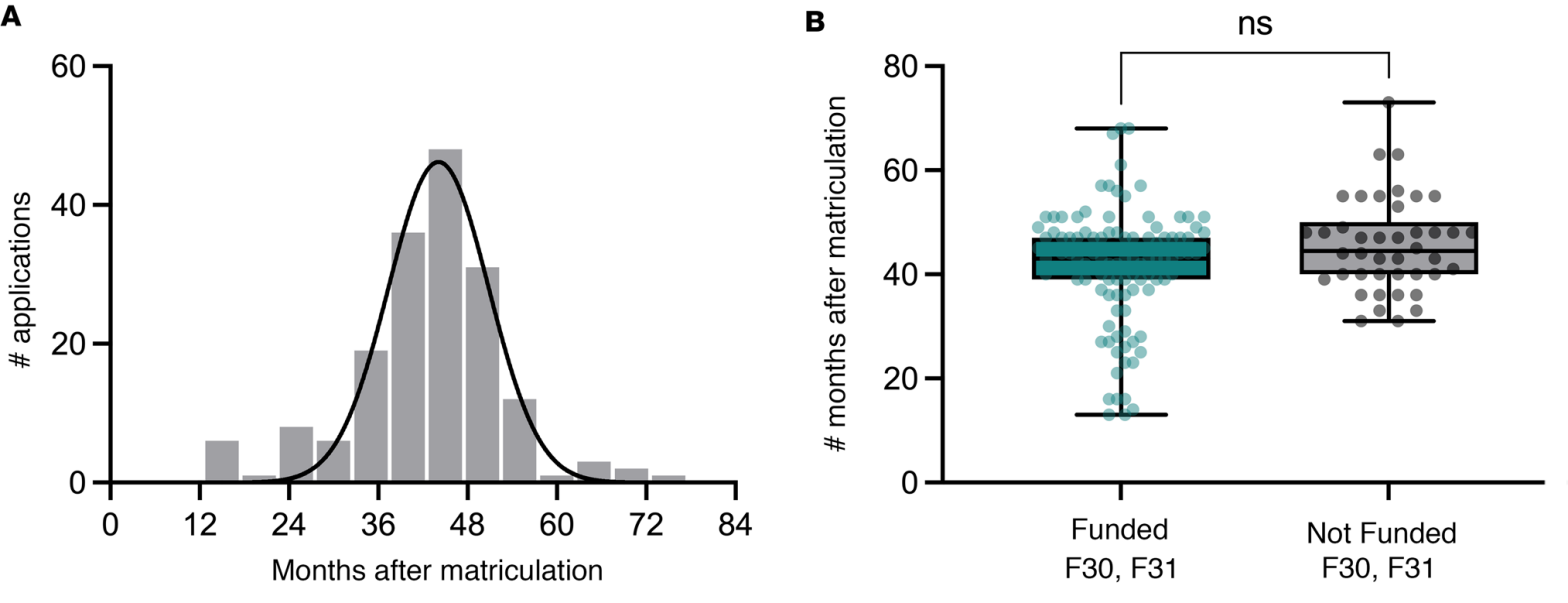
Yale MD-PhD students matriculating 2003–2023 submit F30 applications within the NIH 48-month eligibility window. (**A**) Original submissions submitted after 48 months after matriculation include F31 applications, and F30 applications submitted before 2014, with an average of 43 ± 10 months after matriculation. (**B**) Funded applications were submitted 42 ± 11 months after matriculation; unfunded applications were submitted 46 ± 9 months after matriculation (Kolmogorov-Smirnov test, *P* = 0.32).

In FY2014 (PA-14-150), the NIH restricted the eligibility to apply for F30 funding to applicants who had “matriculated no more than 48 months before the due date of the initial application.” The Notice of Intent to Publish the Reissuance of the Ruth L. Kirschstein NRSA for Individual Predoctoral MD/PhD and Other Dual Doctoral Degree Fellows (Parent F30) Funding Opportunity Announcement (NOT-OD-14-056) justifies the 48-month eligibility period as an effort to encourage “applications from students early in the research training phase of their dual-degree training so that they can substantively benefit from the mentored research training opportunities of an individual fellowship award.” The 48-month eligibility cut-off does not apply to F31 or F31-Diversity applications. Figure 3 describes the distribution of the number of months after matriculation that F30/F31 applications were first submitted, showing that 175 F30 and F31 applications between 2003 and 2022 were submitted an average of 43 months after matriculation. There is no significant difference between when the successful and unsuccessful applications were submitted (Figure 3B; Kolmogarov-Smirnov test, *P* = 0.35). Submissions after 48 months include F30 applications submitted before FY2014 as well as F31 and F31-Diversity applications, which have no limit to submission eligibility.

### Submissions and awards by sex and underrepresented minority group.

Between FY2003 and FY2023, 196 new F30/F31 applications were submitted by 175 unique individuals (some individuals submitted more than one original application) from the Yale MD-PhD Program. Of the 196 new applications, 78 (40%) have been awarded, while ten (5%) are currently pending. In contrast, only 69 applications by 53 unique individuals were submitted to foundations and private charities; of these, 15 (22%) have been awarded.

**Figure 4:**
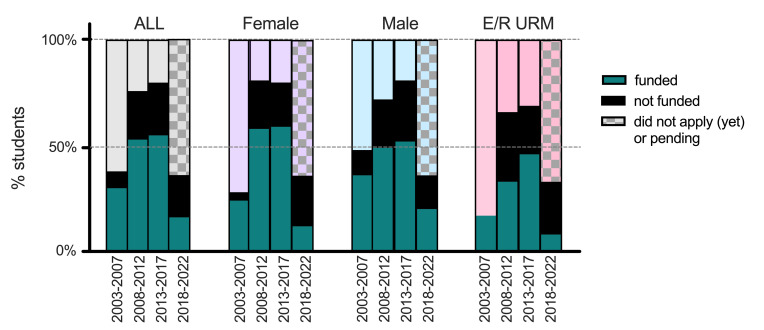
The proportion of students submitting and awarded F30 and F31 applications by sex and underrepresented minority groups. Since 2003, an increasing proportion of Yale MD-PhD students submit F30 and F31 applications; the last 5-year cohort of students who matriculated between 2018 and 2022 are just beginning to prepare and submit NRSA applications. The proportion of funded NRSA (green section of each bar) applications, NRSA applications that were submitted but not funded (black section of each bar), and students who did not submit an NRSA application (top section in light colors) are shown for all, male, female, and ethnic/racial underrepresented minority (E/R URM) students in each 5-year cohort. The patterned section for all the 2018–2022 cohorts include students who have not yet submitted or have submitted applications that are pending.

Figure 4 details the status of F30/F31 applications and awards by all eligible trainees in five-year cohorts (by year of matriculation into the MD-PhD Program), stratified by sex and underrepresented in medicine (URM; NIH-defined ethnic and racial categories) status. The last group (years 2018–2022) includes eligible students who have not yet prepared F30/F31 applications or whose applications were recently submitted and are pending (Figure 4). The proportion of students who submitted applications as well as the proportion of applications funded increased in all cohorts over the 15-year period between 2003 and 2017. Two-way ANOVA analyses comparing the proportions of NRSA-funded males versus females and the proportions of funded URM to non-URM trainees indicate no significant difference based on these demographic stratifications over that 15-year period. Table 1 summarizes the data depicted in Figure 4.

A hidden curriculum is a barrier to success wherein proven strategies for success are taught informally and are not equally accessible to all students (10). The playing field is leveled by requiring all eligible students to apply and providing the necessary resources and critical feedback. In particular, the opportunity to practice skills related to persistence and resilience to feedback and criticism encountered in the application writing and resubmission process is, in the authors’ opinion, invaluable to the future success of students pursuing a career in academic research. Additionally, professionalization and streamlined administration to support students’ fellowship applications are especially important when advancing diversity and inclusivity in research and training capacity.

### Effect of predoctoral NRSA awards on physician-scientist training outcomes.

**Figure 5:**
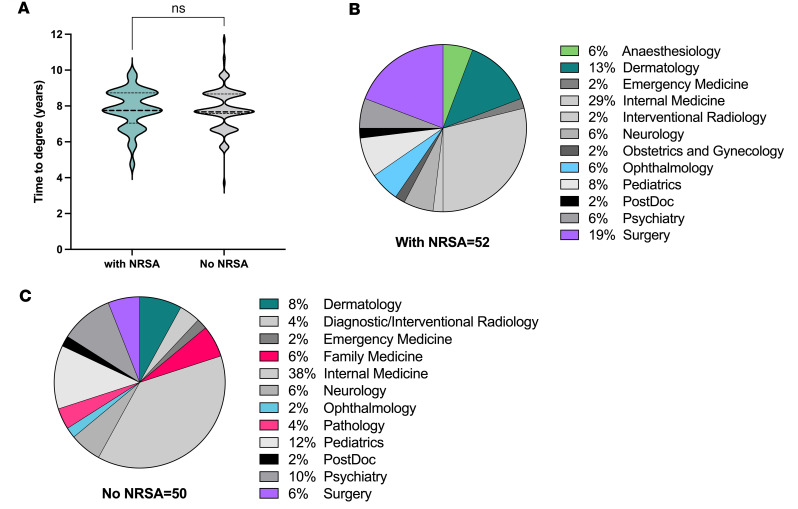
Short-term outcomes. (**A**) The total time to degree is the same for students who held an F30 or F31 during their MD-PhD training and those who did not. (**B** and **C**) Residency disciples of students with NRSA funding (**B**) and without NRSA funding (**C**) reveal slight differences, with funded students preferring highly competitive, procedural specialties such as anesthesiology, dermatology, ophthalmology, and surgery.

For the 288 graduates (through the end of FY2023) who entered the program since 1990, there was no statistically significant effect of having had an F30 or F31 award on the median time to degree, which is 7.8 ± 1.1 years and 7.9 ± 1.2 years with and without an NRSA, respectively (Figure 5A). Six graduates who pursued postdoctoral fellowships rather than residency training did not have NRSA funding. Twenty-six percent (*n* = 76 of 288) were awarded F30 or F31 as MD-PhD trainees.

Of the above 288 graduates, 35% (102 of 288) remain in training as residents or fellows. Of these, 51% (52 of 102) held F30 or F31 awards as program trainees and an additional 8% held other non-NIH predoctoral fellowships. In contrast, only 9% (25 of 186) of all post-GME (all sectors) alumni held F30/ F31 awards; this finding is consistent with the data shown in Figure 1D, indicating that the increases in the number of F30/F31 applications submitted by trainees in the program is relatively recent (see the steep slope that begins in 2012). A comparison of residency choices made by the 102 alumni who remain in training stratified by those who held an NRSA award (Figure 5B) versus those who did not (Figure 5C) revealed that, while individuals within both groups were equally likely to pursue training in internal medicine, neurology, and pediatrics, which are disciplines traditionally chosen by physician-scientists (2), NRSA recipients were more likely to pursue residencies in anesthesiology, dermatology, ophthalmology, and surgery. Moreover, family medicine and pathology residencies were not pursued by NRSA recipients. It is not clear how receipt of an NRSA award affected students’ choices of residency and career interests, and these data do not suggest that NRSA receipt confers success in matching to one residency versus another.

Award of mentored career development (K-type) awards and RPGs are often used to evaluate the long-term outcomes of physician-scientist training (11). Fifty-five of the 288 alumni (19%) of the Yale MD-PhD Program have or have had K awards; of these, only 5 (7%) had been awarded F30/F31 fellowships. Fifty-three of the 288 alumni (18%) have or have had RPGs. Independent of K award success, only 1 alumnus with an RPG had an F30/F31 award. However, 34 alumni with K awards also had subsequent RPG funding. The numbers of alumni who have had both F30/F31 awards and K awards or RPGs are currently not sufficient to determine whether there is a correlation between F30/F31, K, and/or RPG funding success. As has been reported by several groups (11–13), we did find a positive correlation between K award success and subsequent RPG support using Spearman’s correlation (*r*[109] = 0.431, *P* < 0.0001; data not shown) among our alumni. As expected, and shown in Table 2, most of the K award and RPG recipients are currently employed in the academic sector.

## Discussion

The development of scientific writing skills occurs throughout the arc of scientific training, requiring intensive mentorship at every stage. Clear, concise communication of scientific knowledge and its context is learned through drafting and revision of scientific manuscripts and reviews and is exemplified by the dissertation produced by trainees as part of their doctoral training. Research proposals require critical thinking and communication skills, including the ability to formulate and defend a hypothesis, develop primary and alternate research approaches, and outline a rigorous plan for analyzing and interpreting anticipated results. These skills are introduced during the qualifying exam and are honed by the practice of submitting training and research proposals throughout a scientific career. Thoughtful teaching of and mentorship during predoctoral fellowship proposal development provide significant benefits to trainees and institutions. The following strategies have led to increased submissions — itself a desired outcome of our program — as well as improved rates of funding, which benefit trainees, mentors, and our institution.

### Emphasis on training in NRSA fellowship applications.

Thesis advisors are usually skilled at writing research proposals, but their expertise may not translate to successful mentoring of MD-PhD students preparing the comprehensive training plan required for F30/F31 fellowship applications. Our program’s workshops on fellowship preparation specifically emphasize the training aspect of the F30/F31 mechanism and how this affects the scope and type of research proposed. An early and thorough review of draft specific aims eliminates the most common error encountered in F30/F31 applications — an overambitious proposal. For faculty mentors used to writing and reviewing RPGs, the scope of specific aims that are appropriate for an F30/F31 application may seem too limited. Students are also likely to be overly optimistic about the scope and timelines for proposed research aims. We routinely advise students to narrow the scope of the aims, while emphasizing the need for rigorous and detailed descriptions of the experimental approach, careful considerations of pitfalls and alternative approaches, and a realistic timeline that includes both research and clinical training activities as well as new learning experiences. In many cases, students convert edited subaims into alternative approaches or goals, with no negative effect on the rationale and strength of the proposal.

Trainees also must identify new learning experiences that are well integrated into the research plan and will advance their broad professional goal of becoming independent physician-scientists. Such new learning experiences are discussed at every workshop and individual advisory meeting. Coursework that substantially improves a student’s computational or statistical competence is often recommended, as is participation in small, specialized scientific workshops and meetings that expand students’ exposure to diverse professional networks, expertise, and perspectives. Longitudinal or short-term clinical training opportunities during the PhD training years help maintain clinical critical thinking skills while also expanding trainees’ understanding of how patient care and research can be combined within a career.

After peer review, significant individualized advising time with F30/F31 applicants is dedicated to evaluating study section summary statements and preparing a clear and concise introduction to the revised application. Understanding and responding to critical feedback are competencies necessary for every physician-scientist, and the cycle of proposal preparation, review, and resubmission provides multiple opportunities to develop these skills. The resubmission process offers important opportunities to discuss strengthening individual feedback resiliency (14) and maintaining a growth mindset (15) — factors that we believe are critical to the future retention and success of physician-scientists. Students are encouraged to contact NIH program officers as part of this process, and they find them generally very helpful and responsive to questions and requests for guidance. Self-advocacy and communication skills — with their research mentors, collaborators, references, administration, and NIH program officers — are essential for future leadership roles and are acquired throughout the application process.

### Opportunities for strengthening mentor-mentee relationships.

F30/F31 sponsors have varying familiarity with the F30/F31 mechanism and experience with training MD-PhD students. We routinely engage sponsors early in the proposal preparation process and stress the value of a formal training and mentoring plan, as expected in the F30/F31 application, for establishing expectations of both student and advisor during MD-PhD training. In addition to providing written guidelines to students and their mentors for writing the sponsor statement, close attention is paid to first-time mentors of MD-PhD students to ensure that there is sufficient support for the faculty mentor’s professional development as well as the trainee’s. Recommendations include specific guidance on regular meeting times with the student, providing networking opportunities, and in some cases, suggesting a more senior collaborator to be a cosponsor of the F30/F31 application. The inclusion of training activities specifically relevant to physician-scientist careers — such as clinical shadowing and longitudinal electives, which are rarely part of PhD-only training — encourages students and advisors to allocate protected time for these so-called “beyond-lab” experiences throughout the research years.

### Development of writing skills.

Students are encouraged to make use of multiple resources for improving their writing and communication skills. A graduate writing laboratory within Yale University’s Poorvu Center for Teaching and Learning provides writing consultations, workshops, and peer review groups is available to all MD-PhD students. However, we recognize the privileged value of having program faculty with an extensive understanding of the NIH peer review process, having served on or even chaired fellowship study sections, available to advise individual students and their sponsors; this has undoubtedly increased the number of submitted applications. Currently, two faculty advisors are available to support the average of eight students who apply each NIH cycle.

Significant emphasis is placed on developing a clearly written single specific aims page containing all the necessary elements of a successful application: a convincing statement of relevance or impact, strong hypothesis-driven aims, sufficient methodological and statistical detail to address rigor, a simple visual to illustrate the overarching aims, and a statement of how the proposed training plan supports the long-term professional goals of the trainee. Similarly, for applications undergoing resubmission, faculty review general strategies for addressing the critiques of the reviewers in a single-page introduction and individually advise student-mentor dyads on crafting a strong and clear response to the summary statement. Specific guidance is given on appropriate responses such as inclusion of new experiments or preliminary data, clarification of scientific background or rationale, providing additional details (usually on statistics or methods), recruitment of expert collaborators, or identifying new learning experiences. We find that specific subject matter expertise is not necessary to provide insightful feedback for developing strong specific aims and introduction pages, as the overriding goal of these pages is to provide a clear, convincing outline of the detailed research plan that follows.

### Mock study section review to consolidate learning.

The recent implementation of a four-week summer proposal writing module has allowed us to expand upon the concepts covered in the thrice-yearly two-hour workshops. Highly structured instruction and feedback are provided to all participating MD-PhD students on specific aims, rigor and reproducibility of the research plan, and the training plan. The series concludes with a mock study section for which students in the course join our faculty as a study section panel. Previously reviewed F30/F31 applications are discussed (with the permission of the student author), by enrolled students and three faculty preceptors. Applications that were successful upon first submission as well as those that required revision and resubmission are presented. Faculty preceptors joining the study section are given access to the summary statements for each reviewed proposal in advance to help direct the review according to what was discussed in the NIH study section. We find that students astutely apply the review criteria learned in the prior workshops on rigor and training expectations and can replicate the major critiques raised by the actual reviewers of the application.

### Advising around timing of applications.

Eligible MD-PhD students are encouraged to submit primarily F30 applications within the 48-month eligibility period, as these fellowships are designed to support integrated dual-degree training during both graduate research and clinical years. Because there is no eligibility cut-off for F31 or F31-Diversity applications, we generally advise that students reserve the option to apply under the F31 program if the resubmission of the F30 application is unsuccessful. In rare cases, such as when the most appropriate funding institute participates only in an F31 program, trainees have forgone the F30 application. The training, resources, and advice given to MD-PhD students are the same, regardless of the mechanism of funding (F30, F31, or F31-Diversity), with appropriate adjustments to timeline and training plan.

### Building personal and professional skills as a physician-scientist.

By bringing fellowship writing into the MD-PhD Program curriculum, we have significantly lowered the confidence and motivational barriers that can hinder grant submission for trainees. Fostering confidence in grant writing and resilience when receiving critical feedback are invaluable skills required for success in physician-scientist careers and should be developed intentionally and early. By providing training, support, and resources to our students in conjunction with the expectation that all eligible students submit an F30/F31 application, grant writing becomes normalized as a core proficiency to be mastered by all trainees in the program, rather than a daunting and specialized undertaking.

Students gain experience in the time commitment and effort, which are easy to underestimate, required to prepare an effective research proposal. Through the peer review process, they learn how to receive critical feedback well, and to craft thoughtful and effective responses to reviewers’ critiques. This skill of responding to feedback is key to success throughout the trainee’s career, and our faculty advisors spend significant time with each student (and often their sponsor) to prepare a comprehensive and balanced Introduction to the revised application. Discussions often include considerations of feedback resilience and appreciation of reviewers’ critiques as an opportunity for improving research and professional development.

The benefits of preparing a fellowship application are significant and substantial for predoctoral trainees. The opportunity for students to have frank discussions about training expectations, mentor-mentee responsibilities and relationships, balancing clinical and research obligations, rigorous experimental design, professional development activities, and other topics has far-reaching implications for overall trainee preparedness beyond writing the F30/F31 application. Moreover, as the number and success of the F30/F31 applications grows, we hope to see more transitions to successful K awards as trainees improve upon the predoctoral grant writing training. Finally, we anticipate that the increase in confidence and funding success achieved through early training in proposal development will translate to greater retention and success of physician-scientists in the biomedical workforce.

## Methods

We focused on NIH fellowship applications (current funding opportunity announcements for F30, PA-23-260 and PA-23-261; F31, PA-23-272; and F31-Diversity, PA-23-271) submitted by Yale MD-PhD Program trainees between FY2003 and FY2023, limiting our analysis to trainees who have completed or are currently on track to complete the dual degree program. Self-reported demographic data from the Biographic Information section of the American Medical College Application Service (AMCAS) application was used to determine sex and identify students from URM racial and ethnic groups, which includes applicants who select the following racial and ethnic categories: American Indian or Alaskan Nation, Black or African American, Native Hawaiian or Other Pacific Islander, and Hispanic/Latino. Funding information was obtained from the NIH Research Portfolio Online Reporting Tools (RePORT) database (4) and the Yale University Research Enterprise Operations Sponsored Awards database (for predoctoral fellowship application submissions and postaward data from 2003 to present). The FY for a funded fellowship was defined as the date provided in the NIH RePORTER as the actual funding start date, rather than the year the application was submitted. Matriculation year (the year a student entered the MD-PhD program), rather than the year an application was submitted or funded, was used to track individual students. Data on national enrollment of MD-PhD students were obtained from the American Association of Medical Colleges (AAMC) FACTS tables (5, 6, 9).

### Statistics.

Statistical analyses and graphs were produced using GraphPad Prism v9.5.0 for macOS. Where indicated in the text and figures, Kolmogorov-Smirnov test, 2-way ANOVA, or Spearman’s correlation were applied. A *P* value less than 0.05 was considered significant.

### Study approval.

Collection and analysis of data were reviewed by the Yale University IRB and deemed to be exempt under 45CFR46.104(2)(iii).

## Author contributions

BIK and RMF conceived the topic and approach for the study, and together with FSG discussed the results and implications of the data. RMF and FSG offered grant-writing training workshops and provide one-on-one coaching and advice. RMF acquired and analyzed data. All authors contributed to writing and revising the manuscript.

## Supplementary Material

Supplemental data 1

Supplemental data 2

## Figures and Tables

**Table 1:**
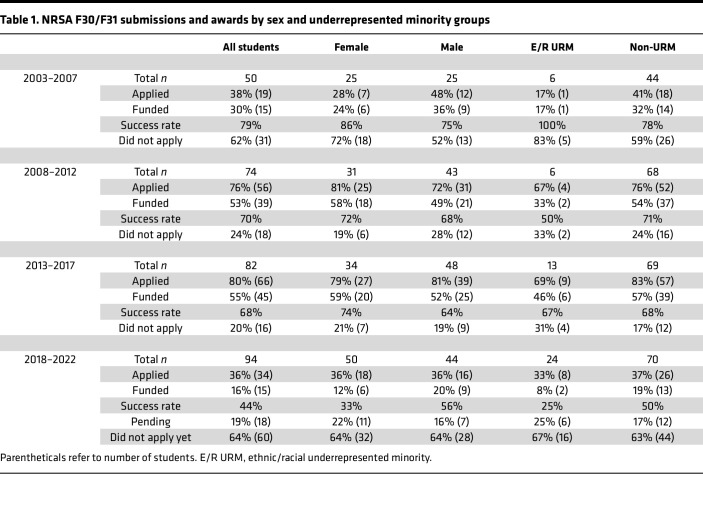
NRSA F30/F31 submissions and awards by sex and underrepresented minority groups

**Table 2:**
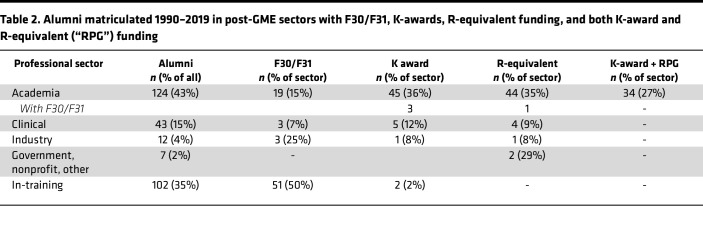
Alumni matriculated 1990–2019 in post-GME sectors with F30/F31, K-awards, R-equivalent funding, and both K-award and R-equivalent (“RPG”) funding

## Version 1 03/08/2024

Electronic publication
