# Supplementary material for: Interventions to support fellowship application success among predoctoral physician-scientists
Source: JCI Insight. 2024 Mar 8;9(5):e175857. doi: 10.1172/jci.insight.175857 (PMC10972582; doi:10.1172/jci.insight.175857)
Supplement: Supplemental data 1 [file jciinsight-9-175857-s122.pdf]

Appendix 1

IRES Pre-submission Survey

Start of Block: Introduction

Q1
This survey is intended to help us ensure that your NIH Ruth Kirschstein National Research Service Awards (NRSA) F30 or F31 fellowship application submission is done as accurately and efficiently as possible. The information you provide here and in your NRSA BOX are required to create and submit a proposal in the IRES Proposal Development (PD) module through the Office of Sponsored Research.

PLEASE NOTE: The entire application MUST be submitted at least 3 days before the NIH deadline so that the Yale Office of Sponsored Projects (OSP) can review the contents and send back any questions or concerns. The application also must be routed to various people, including you and your sponsors, for confirmation before it can be submitted to NIH.

PLEASE COMPLETE THIS SURVEY AT LEAST 2-3 WEEKS BEFORE THE DEADLINE. You will not be able to make an application upload & submission appointment with [redacted] unless these questions are completed.

Section 1 includes some regulatory training and forms that you must complete; signed forms and proof of completion should be uploaded here.

Be sure to consult with your PI if you have any questions about the information requested. Most of the questions are regulatory and/or relate to animal and human subjects.

Submission Appointment slots with [redacted] will not be made available to you until you complete this survey and all required training and forms. Appointment availability is on a first-come, first-serve basis.

The last section of this survey is a checklist of documents that you must prepare for upload to IRES as part of your application. [redacted] will create a folder in BOX for you to put final documents into prior to your appointment to upload the application. You do not have to have all these documents completed to submit this survey.

Please be sure look at a downloadable copy of this survey (https://medicine.yale.edu/mdphd/intranet/grantwriting-guide/Pre-IRES_Survey_416040_51984_v1.pdf) This survey is comprehensive and will be easier to complete once you have all the required materials on hand.

Feel free to reach out to [redacted] or [redacted] for assistance.

Q3 PLEASE BE SURE TO PROVIDE THE BELOW information

- ○ Your eRA Commons ID (3) ____
- ○ PhD Mentor(s) (4) ____
- ○ PhD Department/Program (5) ____
- ○ Your Cellphone # (6) ____

End of Block: Introduction

Start of Block: Research Information

Q4 What is your expected MD-PhD Completion Year (YYYY)?

____

Q5 Primary Location of Research

- ○ Building Name (1) ____
- ○ Room Number (2) ____
- ○ Floor Number (3) ____

Q6 Address of the Laboratory (research site)

- ○ Address 1 (1) ____
- ○ Address 2 (2) ____
- ○ City (3) ____
- ○ State (4) ____
- ○ Zipcode (9-digits) (5) ____

Q7 Field of training for current proposal

▼ 100 Biochemistry (1) ... 989 Other (78)

Q8 Citizenship:

○ US Citizen (1)

○ Non-citizen Permanet Resident (2)

Display This Question:

If Citizenship: = Non-citizen Permanet Resident

Q9 Non-citizen Permanent Resident

○ Permanent US Resident Visa (1)

○ Temporary US Visa (2)

Display This Question:

If Non-citizen Permanent Resident = Temporary US Visa

Q10 If you are a non-US citizen with a Temporary Visa applying for an award that requires Permanent Residency status, do you expect to be granted a Permanent Resident Visa by the start date of the award?

○ Yes (1)

○ No (2)

Q11 Did you attend an MD-PhD Grant-writing workshop (Fred Gorelick, Reiko Fitzsimonds)?

○ Yes (when?) (1) ____

○ No (2)

Q12 Program Announcement (PA) Number

- ◯ PA-23-260 (F30): Ruth L. Kirschstein National Research Service Award (NRSA) Individual Fellowship for Students at Institutions with NIH-Funded Institutional Predoctoral Dual-Degree Training Programs (Parent F30) (1)
- ◯ PA-23-272 (F31): Ruth L. Kirschstein National Research Service Award (NRSA) Individual Predoctoral Fellowship (Parent F31) (2)
- ◯ PA-23-271 (F31-Diversity): Ruth L. Kirschstein National Research Service Award (NRSA) Individual Predoctoral Fellowship to Promote Diversity in Health-Related Research (Parent F31 -Diversity) (3)

Q13 Application Title
(200 characters, including punctuation and spelling)

____

Q14 Proposal Type

- ◯ New (1)
- ◯ Resubmission (2)

Display This Question:

If Proposal Type = Resubmission

Q15 Resubmission: Federal Identifier of your original submission *(can be found on your summary statement*)

____

Q16 NIH Institute

▼ National Cancer Institute (NCI) (1) ... National Library of Medicine (NLM) (21)

Q17 Is this application being submitted to other agencies?

- ◯ Yes (1)
- ◯ No (2)

Display This Question:

If Is this application being submitted to other agencies? = Yes

Q18 What other agencies?

____

Q19 Letters of Reference (3 required; up to 5 allowed).
You should have already reached out to your referees, and provided them with your eRA Commons name, the PA# as well as your specific aims and any other information that can help them write a strong letter. LOR must be submitted by the grant deadline.

- ◯ Referee 1 (Fullname, degree, department, institution) (1) ____
- ◯ Referee 2 (Fullname, degree, department, institution) (2) ____
- ◯ Referee 3 (Fullname, degree, department, institution) (3) ____
- ◯ Referee 4 (Fullname, degree, department, institution) (4) ____
- ◯ Referee 5 (Fullname, degree, department, institution) (5) ____

Q20 Will the proposed studies generate large-scale human or non-human genomic data as detailed in the NIH Genomic Data Sharing Policy?
(see the NIH Grants Policy Statement, Section 2.3.7.10: NIH Genomic Data Sharing and

Section 8.2.3.3: Genomic Data Sharing (GDS) Policy/Policy for Genome-Wide Association Studies (GWAS)).

- ◯ Yes (1)
- ◯ No (2)

Q21
OPTIONAL
Assign to Study Sections

See link https://public.csr.nih.gov/StudySections/Fellowship, and consult with your PI and Program Officer.

- ◯ First Choice (1) ____
- ◯ Second Choice (2) ____
- ◯ Third Choice (3) ____

Q22
OPTIONAL
Expertise: *only 40 characters allowed*
Identify scientific areas of expertise needed to review your application.
This is important if your research is particularly specialized.

- ◯ 1 (1) ____
- ◯ 2 (2) ____
- ◯ 3 (3) ____
- ◯ 4 (4) ____
- ◯ 5 (5) ____

Q23 Current or prior Kirschstein-NRSA support (including MSTP T32 support). A total of 6 years of NIH training support is allowed. Alex uses this information to create your budget request.

Instructions to screenshot current/past support from your eRA Commons page.
1) Login to eRA Commons
2) Select *xTRAIN*

3) Select *Appointments & Terminations* tab
4) Screenshot your support and upload here

Q24 Proposed Budget Period

The start date is an estimate, and is typically at least nine months after application submission. The project period should not exceed what is allowed in the FOA.

The budget will be calculated and entered based on these dates.

○ Project Start Date (mm/dd/yyyy) (1) ____

○ Project End Date (mm/dd/yyyy) (2) ____

*

Q25 Short proposal title (limit 30 char)
For internal Yale use only

____

Q26 Sponsor

○ Name (1) ____

○ eRA Commons ID (2) ____

○ Joint VA Appointment? (8) ____

Q27 Co-sponsor (if applicable)

○ Name (1) ____

○ eRA Commons ID (2) ____

○ Joint VA appointment? (4) ____

Q28 Optional: Do you have a collaborator1 with significant contribution to the application (applicable if s/he is responsible for experimental design, *biosketch needed)

- ○ Name (1) ____
- ○ eRA Commons ID (2) ____
- ○ Joint VA appointment? (4) ____

Q29 Optional: Do you have a collaborator2 with significant contribution to the application (applicable if s/he is responsible for experimental design, *biosketch needed)

- ○ Name (1) ____
- ○ eRA Commons ID (2) ____
- ○ Joint VA appointment? (4) ____

End of Block: Research Information

Start of Block: REGULATORY REQUIREMENTS

Q30

REGULATORY REQUIREMENTS: UPLOADS REQUIRED

Upload signed Yale University NRSA Assurance of Compliance Form
Click HERE to download the form which must be signed by you (applicant) and your PI. (https://your.yale.edu/sites/default/files/1304_fr.03c_national_research_service_award_nrsa_assurance_of_compliance_0.pdf)

*If this is a resubmission, please be sure to update the dates on the form.

Upload required.

Q31
Yale Conflict of Interest Disclosure Form

Go to https://your.yale.edu/research-support/conflict-interest; upon completion, please click on the PDF icon to save a copy of your disclosure and upload it here.

Upload required.

Q32

Sponsored Projects Administration Training

Go to https://bmsweb.med.yale.edu/tms/tmspage and enter "Sponsored Projects Administration for Faculty" in the keyword search.

This information is important, and detailed. Please take the time to read it. You must pass the quiz at the end.

Please take a screenshot of your passing score (>90%) and upload it here.

Upload required.

Q33

Patent Policy Acknowledgement Agreement

Go to https://bmsweb.med.yale.edu/tms/tmspage and enter "Patent Policy Acknowledgement Agreement" in the keyword search.

Please take a screenshot that you have completed the course and upload it here.

Upload required.

Q34 ORCID: The *Open Researcher and Contributor ID* (ORCID) ID is used within NIH and Grants.gov to relate publications to grants. You must associate your ORCID ID to your eRA Commons personal profile.

STOP here until you have successfully linked your populated ORCID ID to your Commons account and populated the ORCID with publications and other details.

I have successfully linked my populated ORCID to my Commons account.

- ◯ Yes (4)
- ◯ No (5)

End of Block: REGULATORY REQUIREMENTS

Start of Block: Proprietary

Q35 Is proprietary/privileged information included in the application?

Patentable ideas; trade secrets; or privileged, confidential commercial, or financial information should be included in applications only when such information is necessary to convey an understanding of the proposed project. If the application includes such information, check “Yes” and clearly mark each line or paragraph on the pages containing the proprietary/privileged information with a statement similar to: “The following contains proprietary/privileged information that (name of applicant) requests not be released to persons outside the government, except for purposes of review and evaluation.” This statement can be included at the top of each page as applicable.

○ Yes (1)

○ No (2)

Q36 Does this Project Have an Actual or Potential Impact - positive or negative - on the environment?

If an applicant expects that the proposed project will have an actual or potential impact on the environment, or if any part of the proposed research and/or project includes one or more of the following scenarios, check “Yes.”
1. The potential environmental impacts of the proposed research may be of greater scope or size than other actions included within a category.
2. The proposed research threatens to violate a federal, state, or local law established for the protection of the environment or for public health and safety.
3. Potential effects of the proposed research are unique or highly uncertain.
4. Use of especially hazardous substances or processes is proposed for which adequate and accepted controls and safeguards are unknown or not available.
5. The proposed research may overload existing waste treatment plants due to new loads (volume, chemicals, toxicity, additional hazardous wasted, etc.).
6. The proposed research may have a possible impact on endangered or threatened species.
7. The proposed research may introduce new sources of hazardous/toxic wastes or require storage of wastes pending new technology for safe disposal.
8. The proposed research may introduce new sources of radiation or radioactive materials. 9. Substantial and reasonable controversy exists about the environmental effects of the proposed research.

○ Yes (1)

○ No (2)

Display This Question:

If Does this Project Have an Actual or Potential Impact - positive or negative - on the environment?... = Yes

Q37 If yes, please explain (limit 55 characters)
You may cut-and-paste here, or have a document in BOX with these responses ready to cut-and-paste into IRES.

____

Display This Question:

If Does this Project Have an Actual or Potential Impact - positive or negative - on the environment?... = Yes

Q38 If this project has an actual or potential impact on the environment, has an exemption been authorized or an environmental assessment (EA) or environmental impact statement (EIS) been performed?

- Yes (1)
- No (2)

Display This Question:

If If this project has an actual or potential impact on the environment, has an exemption been autho... = Yes

Q39 If yes, please enter additional details about the EA or EIS (limit 55 characters)
You may cut-and-paste here, or have a document in BOX with these responses ready to cut-and-paste into IRES.

____

Q40 Is the research performance site designated, or eligible to be designated, as a historic place?

- Yes (1)
- No (2)

Q41 Does this project involve activities outside of the United States or partnerships with international collaborators?

○ Yes (1)

○ No (2)

Display This Question:

If Does this project involve activities outside of the United States or partnerships with internatio... = Yes

Q42 If yes, identify the countries (limit 55 characters; you may use abbreviations)
You may cut-and-paste here, or have a document in BOX with these responses ready to cut-and-paste into IRES.

____

Display This Question:

If Does this project involve activities outside of the United States or partnerships with internatio... = Yes

Q43
If you checked "Yes" to international collaborators, you must write a "Foreign Justification" for upload to IRES.

Your justification must include a description of how the foreign training is more appropriate than in a domestic setting. Include reasons why the facilities, the sponsor, and/or other aspects of the proposed experience are more appropriate in a foreign setting. The justification is evaluated in terms of the scientific advantages of the foreign training as compared to the training available domestically. Foreign training will be considered for funding only when the scientific advantages are clear.

File upload at this time is OPTIONAL, but please have it ready for submission at the time of your appointment!

End of Block: Proprietary

Start of Block: HUMAN SUBJECTS

Q44
HUMAN SUBJECTS

Will your proposal involve the use of human subjects?

If activities involving human subjects are planned at any time during the proposed project at any performance site, check “Yes.” Check “Yes” even if the proposed project is exempt from regulations for the Protection of Human Subjects, or if activities involving human subjects are anticipated within the period of award but plans are indefinite.

Need help determining whether your application includes human subjects? Check out the NIH Research Involving Human Subjects website for information, including a Human Subjects Decision Tool designed to walk applicants through the decision process. Note on the use of human specimens or data: Applications involving the use of human specimens or data may or may not be considered to be research involving human subjects, depending on the details of the materials to be used.

○ Yes (1)

○ No (2)

Display This Question:

If HUMAN SUBJECTS Will your proposal involve the use of human subjects? If activities involving hu... = Yes

Q45 Because your project involved human subjects, additional detailed information is required to complete submission of your application. **Please be sure to review the following questions in consultation with your PI so that you provide the necessary information correctly.**

Display This Question:

If HUMAN SUBJECTS Will your proposal involve the use of human subjects? If activities involving hu... = Yes

Q46 Human Subjects: Protocol Approval Status and Date

Students are usually listed on their PI's IRB protocols; you do not need to submit your own

applications to the IRB. Please consult with your PI regarding submission/approval of these protocols.

☐ IRB Approval Pending (Applicants should check this box even if the IRB review/approval process has not started by the time of submission) (1)

☐ IRB Approval Date (Leave blank if IRB approval is pending. An IRB approval date is not required at the time of submission) (2) ____

☐ IRB Approval Number (3) ____

Display This Question:

If HUMAN SUBJECTS Will your proposal involve the use of human subjects?   If activities involving hu... = Yes

Q47 Is the Project Exempt from Federal regulations?

If the project is exempt from federal regulations, check “Yes” and check the appropriate exemption number. Human subjects research should only be designated as exempt if all of the proposed research projects in an application meet the criteria for exemption. If the project is not exempt from federal regulations, check “No.” For more information, see the [NIH’s Exempt Human Subjects Research infographic]*.*

○ Yes (1)

○ No (2)

Display This Question:

If HUMAN SUBJECTS Will your proposal involve the use of human subjects?   If activities involving hu... = Yes

Q48 If yes, select the appropriate exemption number 1, 2, 3, 4, 5, 6, 7, 8:

Need help determining the appropriate exemption number? Refer to NIH's [Research Involving Human Subjects Frequently Asked Questions]*.*

▼ - (1) ... 8 (9)

Display This Question:

If HUMAN SUBJECTS Will your proposal involve the use of human subjects? If activities involving hu... = No

Q49 If no to human subjects, does the proposed research involve human specimens and/or data?

○ Yes (1)

○ No (2)

Display This Question:

If If no to human subjects, does the proposed research involve human specimens and/or data? = Yes

Q50 Provide an explanation for why the application does not involve human subjects research even though it involved human specimens and/or data.

Not Human Subjects Research Description
Under some circumstances, research involving only unidentifiable/de-identified or coded private information or biological specimens is not human subjects research because investigators cannot readily ascertain the identities of the individuals to whom the data or samples belong. In such cases, IRB review is not required. ***The PI makes and certifies this determination****.*

In order for your use of data and/or biological specimens to not meet the definition of a human subject, all of the following conditions must apply: The research is not FDA-regulated.
The research team will not have access to identifiers or keys to link coded data (even temporarily).
You are not conducting human stem cell research.
Note: If the project is not human subjects research, it does not matter whether or not the information or specimens existed or are collected before the study is proposed.
You may cut-and-paste here, or have a document in BOX with these responses ready to cut-and-paste into IRES.

____

____

____

Q51 Will your proposal involve human embryonic stem cells?

- Yes (1)
- No (2)

Display This Question:

If Will your proposal involve human embryonic stem cells? = Yes

Q52 Cell Line(s): List the 4-digit registration number of the specific cell line(s) from the NIH hESC Registry (e.g. 0123). Up to 200 lines can be added.

____

Q53 Is this proposal funding a clinical trial?

- Yes (1)
- No (*typical answer) (2)

Q54 Is this proposal an NIH-defined phase 3 clinical trial?

- Yes (1)
- No (*typical answer) (2)

End of Block: HUMAN SUBJECTS

Start of Block: ANIMAL SUBJECTS

Q55
ANIMAL SUBJECTS

Will your proposal involve the use of laboratory (vertebrate) animals?

If activities involving vertebrate animals are planned at any time during the proposed project at any performance site, check “Yes.” Otherwise, check “No”. Note that the generation of custom

antibodies constitutes an activity involving vertebrate animals. If animal involvement is anticipated within the period of award but plans are indefinite, check "Yes."

- ◯ Yes (1)
- ◯ No (2)

Display This Question:

If ANIMAL SUBJECTS Will your proposal involve the use of laboratory (vertebrate) animals? If activ... = Yes

Q56 Animal Subjects: Protocol Approval Status and Date

Students are usually listed on their PI's IACUC protocols; you do not need to submit your own applications to the IACUC. Please consult with your PI regarding submission/approval of these protocols.

- ▢ IACUC or IRB Approval Pending (Applicants should check this box even if the IACUC/IRB review/approval process has not started by the time of submission) (1)
- ▢ IACUC Approval Date (Leave blank if IACUC approval is pending. An IACUC approval date is not required at the time of submission) (2) ____
- ▢ IACUC Approval Number (3) ____

Display This Question:

If ANIMAL SUBJECTS Will your proposal involve the use of laboratory (vertebrate) animals? If activ... = Yes

Q57 Are vertebrate animals euthanized?

- ◯ Yes (1)
- ◯ No (2)

Display This Question:

If Are vertebrate animals euthanized? = Yes

Q58 If “Yes” to euthanasia, is method consistent with AVMA guidelines?

For more information: See AVMA Guidelines for the Euthanasia of Animals.

- ◯ Yes (1)
- ◯ No (2)

Display This Question:

If Are vertebrate animals euthanized? = No

Q59 If “No” to AVMA guidelines, you must describe (in 1000 characters or fewer) the method of euthanasia and provide a scientific justification for its use.
You may cut-and-paste here, or have a document in BOX with these responses ready to cut-and-paste into IRES.

____

Display This Question:

If ANIMAL SUBJECTS Will your proposal involve the use of laboratory (vertebrate) animals? If activ... = Yes

Q60
Write a “Vertebrate Animals” attachment for upload to IRES if you answered “Yes” to the question “Are Vertebrate Animals Used?”

An example of a VERTEBRATE ANIMALS Attachment can be found HERE (https://yale.box.com/s/l3dzkvkyx83qsih7r8ub7v78cawly8p1)

File upload at this time is OPTIONAL, but please have it ready for submission at the time of your appointment!

End of Block: ANIMAL SUBJECTS

Start of Block: Yale Regulatory Questions

Q61

OTHER YALE REGULATORY QUESTIONS

Please read carefully, and consult with your PI or Lab manager if you have any questions.

EXPORT CONTROLS

In the interest of protecting the national security, foreign policy and economic interests of the U.S., the federal government regulates the transfer of information, commodities, technology, and software which are of strategic importance to the U.S. As a result, there is a complex body of laws collectively referred to as “Export Controls.” Generally, Export Controls regulate the shipment or transfer, by whatever means, of controlled items, software, technology, or services out of U.S. (termed an “Export”). In addition, these regulations also restrict the release of certain information to foreign nationals here in the U.S. (referred to as a “Deemed Export”).

In order to ensure compliance with Export Controls, applicants need to identify when their activities may trigger such regulations. A positive answer to one of the following questions could destroy the Fundamental Research Exclusion (FRE), which is an exception to Export Control restrictions. The FRE generally applies to basic and applied research in science and engineering when the resulting information is shared broadly within the scientific community. The FRE applies only to the dissemination of research data and information, not to the transmission or shipment of material goods or equipment, which remain subject to Export Controls regardless of the applicability of the FRE. If you accept confidential or proprietary information subject to a Confidentiality or Non-Disclosure Agreement (NDA), and the disclosure restrictions affect your ability to publish research results, the research itself will lose its characterization as 'fundamental research' for Export Control purposes. To preserve the applicability of the FRE to otherwise fundamental research, NDAs and similar confidentiality agreements must be entirely peripheral to the research program (i.e., sufficiently remote from the intellectually significant portions of the research), and the disclosure restriction must not affect the ability to publish the research results.

Guidance on Export Controls and Electronic Devices in International Travel

NOTE:Transactions / activities involving individuals or entities in / from the following countries and territories are comprehensively embargoed by OFAC and are generally PROHIBITED, absent prior review and approval from Yale's Office of Export Controls - Cuba, Iran, North Korea, Crimea Region (Russia-Ukraine), or Syria.

Please contact Donald Deyo, Director, Export Controls, Senior Advisor Contracts prior to submitting any proposed sponsored project that involves individuals or entities in / from these countries: donald.deyo@yale.edu.

Other countries, while of lesser concern, may be subject to targeted sanctions which could impact your project. All countries subject to any OFAC sanctions are identified by an asterisk in

the dropdown menu.
Country List: Balkans (Albania, Bosnia, Croatia, Macedonia including Kosovo, Serbia), Belarus, Burundi, Central African Republic, Congo, Iraq, Lebanon, Libya, Nicaragua, Somalia, South Sudan, Russia-Ukraine, Venezuela, Yeman and Zimbabwe.

Q62
Does the proposed sponsored project, discussion with the Sponsor or the proposed award document refer to or require any of the following:

	Yes (1)	No (2)
*1a. Export Controls in general or receipt of Export Controlled materials / software / information, such as through a non-disclosure agreement (1)	○	○
*1b.Publication Restrictions (2)	○	○
*1c. Restrictions on foreign national from participating in the proposed sponsored project (3)	○	○

Q63
Does the proposed sponsored project involve any interaction in a foreign country, or with a foreign entity or foreign national (at Yale or outside the U.S.)?

○ Yes (1)

○ No (2)

Q64
Will the proposed sponsored project involve the transfer or shipment of equipment, materials,

software, or data, or the provision of services, outside the U.S.?

◯ Yes (1)

◯ No (2)

Q65
Does the proposed sponsored project involve any of the following?

	Yes (1)	No (2)
*4a.Sponsorship by DOD, DHS, DOE, NASA, NIST (1)	◯	◯
*4b. Any technology or software which involves encryption, possible military applications or the possibility to use such technology in development of weapons (2)	◯	◯

Q66
Does the proposed sponsored project involve the use of any Controlled Un-Classified Information?

◯ Yes (1)

◯ No (2)

Q67
Are EHS materials used in the proposed research?

	Yes (1)	No (2)
* Recombinant DNA (1)	○	○
* Hazardous Chemicals (2)	○	○
* Radioactive Materials/Sources (7)	○	○
* Select Agents (8)	○	○
* Human Gene Transfer (9)	○	○
* Controlled Substances (10)	○	○
* Radiation Generating Equipment (11)	○	○
* Biohazards (12)	○	○
* Class 3b or 4 Lasers (13)	○	○
* Human Pathogens (14)	○	○
* Human Embryonic Stem Cells (15)	○	○

Q68 Subrecipient Conflict of Interest: If the prime sponsor is a PHS agency, a PHS-like sponsor or the NSF, does the subrecipient institution have a PHS/NSF Compliant COI?

○ Yes (1)

○ No (2)

Display This Question:

If Subrecipient Conflict of Interest: If the prime sponsor is a PHS agency, a PHS-like sponsor or t... = No

Q69 Enter the name and role of the individual(s) identified by the subrecipient as being responsible for the design, conduct or reporting of the research.

- ○ Individual Name (1) ____
- ○ Subrecipient Institution (8) ____
- ○ Role (2) ____

Q70
Does this research project involve the collection of genomic data?

- ○ Yes (1)
- ○ No (2)

Display This Question:

If Does this research project involve the collection of genomic data? = Yes

Q71 Is there a plan for the submission and subsequent sharing of such data?
***NOTE: If No, then a justification for any data submission exceptions must be included in the proposal budget.*
If Yes, related costs of such a plan must be included in the proposal budget.

- ○ Yes (1)
- ○ No (2)

End of Block: Yale Regulatory Questions

Start of Block: DOCUMENTS

Q72
BIOSKETCHES

Your biosketch and your sponsor(s)' biosketch MUST contain the correct information and formatting. This is one of the major sources of error that stall submission through Yale OSP. Please be sure to upload the biosketches in your BOX folder as soon as possible so that Alex can check the formatting and information.

Do NOT assume that your sponsor's Biosketch is correct--you must check to make sure that it is on the correct NIH form, that it is within the 5-page limit, that there are only 4 publications in the personal statement, only 5 "Contributions to Science" and that only on-going and completed research projects from the past 3 years is listed. *N.B. The Other Support page requires a PI signatures, so BE SURE that your sponsor(s) is available to help you with a NEW BIOSKETCH early. Do not leave it to the last minute!!

Be sure to use the correct form pages and formatting! Follow format and instructions EXACTLY according to https://grants.nih.gov/grants/forms/biosketch.htm

File upload at this time is OPTIONAL (please combine all biosketches into one PDF), but please have it ready for submission at the time of your appointment!

Q73 **MAIN SECTIONS OF THE FELLOWSHIP APPLICATION**
UPLOADS: This section is for uploading the project summary/abstract, project narrative, references, information on facilities, and equipment lists (templates are available). These sections should be completed and uploaded PRIOR to the second grantwriting workshop. Sections related to the project may be revised and re-uploaded at the time of your submission appointment with Alex.

All uploaded documents should comply with the formatting requirements: 0.5" margins -- ALL (top, bottom, left, right) 11 point font, Arial preferred

PLACE THE WORD AND PDF DOCUMENTS (PROPERLY NAMED, SEE CHECKLIST) IN YOUR NRSA FOLDER IN BOX.

You may check these documents off here as you complete them.

- ☐ PROJECT SUMMARY (limit 30 lines of text in Word) (4)
- ☐ PROJECT NARRATIVE (3 sentences) (5)
- ☐ BIBLIOGRAPHY & REFERENCES CITED (6)
- ☐ TRAINING in the RESPONSIBLE CONDUCT OF RESEARCH (7)
- ☐ FACILITIES & OTHER RESOURCES (8)
- ☐ EQUIPMENT (9)
- ☐ DESCRIPTION OF INSTITUTIONAL ENVIRONMENT & COMMITMENT TO TRAINING (1 page template) (10)
- ☐ ADDITIONAL EDUCATION INFORMATION (1 page template) (11)
- ☐ RESOURCE SHARING PLAN (12)

End of Block: DOCUMENTS

Start of Block: END

Q74
This is the end of the pre-IRES submission questionnaire. If you are certain of all your responses, please hit the next arrow below to SUBMIT.
\
You will receive an email shortly with a link to the SIGNUP GENIUS to set up your submission appointment with Alex.

End of Block: END
