# Supplementary material for: Interventions to support fellowship application success among predoctoral physician-scientists
Source: JCI Insight. 2024 Mar 8;9(5):e175857. doi: 10.1172/jci.insight.175857 (PMC10972582; doi:10.1172/jci.insight.175857)
Supplement: Supplemental data 2 [file jciinsight-9-175857-s123.pdf]

Appendix 2

NRSA Intent to Apply

Start of Block: Block 1

Q1 In which cycle do you intend to submit?

- ○ April 8 (1)
- ○ August 8 (2)
- ○ December 8 (3)

Q2 What type of NRSA will you be submitting? Please be sure to check your eligibility.

- ○ F30 (1)
- ○ F31 (4)
- ○ F31-Diversity (5)
- ○ Not sure (7)

Q3 Is this a new submission or resubmission?

- ○ NEW submission (1)
- ○ Resubmission (2)

Display This Question:

If Is this a new submission or resubmission? = Resubmission

Q7 Please upload or email your summary statements to Drs. [redacted] and arrange to discuss your resubmission with them. *These summary statements are kept fully confidential; reviewer comments are often helpful for us to understand how to provide the best advice to you and future applicants from our program. They will not be shared with anyone else.*

Q8 A human subject is "a living individual about whom an investigator is conducting research including the following:

- Obtains information or biospecimens through intervention or interaction with the individual, and uses, studies, or analyzes the information or biospecimens; or
- Obtains, uses, studies, analyzes, or generates identifiable private information or identifiable biospecimens."

Will you have human subjects in your research proposal?

◯ Yes (1)

◯ No (2)

◯ Not sure (3)

Display This Question:

If A human subject is "a living individual about whom an investigator is conducting research includi... = Yes

And A human subject is "a living individual about whom an investigator is conducting research includi... = Not sure

Q9 Use this NIH Human Subjects Decision Tool https://grants.nih.gov/policy/humansubjects/hs-decision.htm to assist you with determining if your research involves human subjects, may be considered exempt from Federal regulations, or is not considered human subjects research

What did the Decision Tool conclude?

◯ Yes, it is human subjects research (4)

◯ It is exempt human subjects research (under which exemption? see https://grants.nih.gov/sites/default/files/exemption_infographic_v8_508c_1-15-2020.pdf) (7) ____

◯ No, it is not human subjects research (5)

Q5 Your sponsor is responsible for working closely with you to develop your training and research plan, to review your drafts and to provide a strong SPONSOR LETTER. Please be sure that your sponsor(s) has the time needed for you to get their input and guidance.

Who is your Sponsor? If you will have a co-sponsor, please enter here.

- ○ Sponsor Lastname (1) ____
- ○ Sponsor Firstname (4) ____
- ○ Sponsor email (2) ____
- ○ Co-sponsor Name (enter NONE if not applicable) (3) ____

End of Block: Block 1
